# Whither social determinants of health?

**DOI:** 10.11606/s1518-8787.2020054001618

**Published:** 2020-01-23

**Authors:** Fúlvio Borges Nedel, João Luiz Bastos

**Affiliations:** I Universidade Federal de Santa CatarinaDepartamento de Saúde PúblicaFlorianópolisSCBrasil Universidade Federal de Santa Catarina. Departamento de Saúde Pública. Florianópolis, SC, Brasil

**Keywords:** Social Determinants of Health, Public Health, Health Status Disparities, Social Inequity, Research

## Abstract

This critical commentary extends the debate on social determinants of health and disease. Its main argument is that while further studies are unnecessary to demonstrate the fundamentally social distribution of health outcomes, extant analyses rarely engage with the fact that poverty and other forms of oppression are political choices made by societies, which are both contemporaneously contingent and historically situated. This view must guide research and debate in the area so that studies intending to bring injustice to light do not end up naturalizing it. Research based on this fundamental understanding may help to overcome the narrow scope of multicausal black box approaches, which do not analyze the interrelations among determinants and make only a limited contribution to the construction of healthy societies.

## Whither social determinants of health?

The notion that health is socially determined, i.e., that one’s health depends on the society in which one lives, is not new, and can be found in Hippocratic texts, such as *On Airs, Waters and Places*. This idea re-emerged in the Western world with Ramazzini’s work on *Diseases of Workers *at the end of the 17^th^ century. In the first half of the 19^th^ century, through the work of Alexandre-Louis, Villermé, Engels, and especially the social medicine movement by Virchow and others, knowledge was constructed around the idea that people’s (and consequently populations’) health, disease and death depend on living conditions, which depend, in turn, on the social conditions of reproduction of life^1^.

Notably, such knowledge does not strictly assume identification of an etiological agent of disease—and few associations will be as strong as those reported by Villermé that half of the employers’ children reached the age of 21, while half of the workers’ children died before the two years of age. Thinking on this topic co-exists with two closely related issues: (1) the wish to find simple explanations for some health issues, such that “each disease has a single cause,” more or less defined—an explanation that is still sought, especially within molecular and genetic epidemiology of the past few decades^2^; and (2) a fundamental ideological debate that dates back at least to Chadwick^3^ centered on whether we should change the social organization to promote health.

Thus, in the midst of growing capitalism, the advent of bacteriology at the end of the 19^th^ century allowed proposals to transform cities and societies, presented by supporters of miasmatic theories^3^, to be ignored. We could say that when we cleansed ourselves from miasmatic theories, the capitalism of that period took the opportunity to throw out, together with the bathwater, the social determinants of health ‘baby’. Understanding that health is socially determined brings with it an imperative of social change, in order to improve the population’s health standards. For a society with fewer unjustifiable health inequalities to be realized, a society with fewer social injustices must be created. To do so, progress in biomedical knowledge is necessary, but insufficient^4^. We then have an ethical issue that is equally essential. The problem is more than simply the existence of health differentials across population subgroups. They will always exist, since people and societies are different, and epidemiology or public health should not be the instruments of cultural homogenization; our focus is on unfair health inequalities^5^.

In this sense, Winslow’s definition of public health is a remarkable achievement at the beginning of the 20^th^ century^6^, a time when the unicausal explanation of disease was hegemonic, although tempered by the conception of the “ecological triad.”^7^ Bacteriologist and founder of the Yale School of Public Health in the USA, Winslow proposed one of the most cited definitions of public health to date. In a publication in which the author discusses the multidisciplinary character of public health, in addition to the broad attributions of its professionals, Winslow risks the following definition: “Public health is the science and the art of preventing disease, prolonging life, and promoting physical health and efficiency through organized community efforts for the sanitation of the environment, the control of community infections, the education of the individual in principles of personal hygiene, the organization of medical and nursing service for the early diagnosis and preventive treatment of disease, and the development of the social machinery which will ensure to every individual in the community a standard of living adequate for the maintenance of health^6^.”

This definition not only conceptualizes public health as a branch of science concerned with health—not disease—, but also recovers the notion and rhetoric found in the *Manifesto of Social Medicine* by Virchow that society can organize itself to ensure a standard of living compatible with the maintenance of good health^8^. It also relies on a sharp distinction between concepts that have been, only in subsequent decades, widely debated and refined in the area, such as disease prevention and health promotion^9^. Winslow connects population patterns of health, disease and well-being with aspects of the social, economic and political arenas: the health services and social machinery to which the author refers. We risk arguing, however, that this definition has gained popularity more due to its bacteriological dimension—which allowed the Establishment to emphasize individuals’ disease, as well as the understanding that health promotion is only part of disease prevention—than to its attempt to highlight the social determinants of health.

Thus, despite definitions such as Winslow’s, the re-emergence of theoretical perspectives favoring a relationship of dependence between forms of social organization and individuals’ health co-existed with a reiteration of biomedical thinking on the origins of health problems. From the second half of the 20^th^ century onwards, amid the epidemiological transition and after the experience of two world wars, Nazism and fascism, the Marshall Plan and McCarthyism^10^, black box epidemiology became hegemonic^11^. This causal reasoning, which is first portrayed as multicausal, reveals itself as unicausal in practice by explaining the function of each causal factor as if acting alone, in a simplistic analysis that neglects relationships among them, as well as their underlying causes (the “causes of causes”^12^)^13^. Decontextualized multicausalities (or “webs without spiders” – that is, webs of causation in which fundamental causes are not depicted, in Nancy Krieger’s words^13^) that had been developed decades before gained traction and countless advocates. These models eventually became the hegemonic approach to explaining the origin of diseases and to proposing strategies to address them. Within these biomedical perspectives, the emphasis was on clinical characteristics (high cholesterol, arterial hypertension, sedentary lifestyle etc.), which were taken as the root causes of health and disease, presented as if floating in a social vacuum, with no connection to historiographic accounts. Social determinants of health have a minor role in analyses drawing from biomedical perspectives, regardless of the statistical significance they might reach in multilevel or structural equation models.

Theoretical and empirical works demonstrating the insufficiency of black box epidemiology^14-16^ are noteworthy. Such insufficiency is not only due to a reliance on a strict biomedical approach, but also stems from a lack of effort to develop more complex analyses that consider the relationships among causal factors, as well as their determinants, at least in the theoretical model of the study. This means that behavioral and related approaches that eventually include indicators of social conditions or “contextual factors” in the analysis will also be insufficient if they ignore this fundamental flaw.

Clashes between these distinct styles of public health thinking^17^—especially those reflecting the dissonance between more positivist approaches and Winslow’s definition of public health^6^—cause consternation among some scholars. Building on an imaginary court, Shy^18^ portrays himself as “witness for the prosecution” in a 1997 article, arguing that epidemiology has failed to achieve the goals of public health. Amid this scenario, he criticized the hegemonic biomedical thinking in epidemiology, as well as the excessive emphasis on “micro” and mostly clinical factors, which hardly contribute to effective interventions to improve the populations’ health. Such emphasis, coupled with social and historical decontextualization, has limited the study of political, economic and cultural forces that determine population patterns of health and disease^4,18^.

This all happens, however, hand in hand with an increase in publications^19^ and in the number of research groups focused on social inequalities in health and social determinants of health, especially in the past two decades. A significant increase in publications^19^ providing greater weight to a particular type of scientific knowledge that, as mentioned above, was already well-established, occurred in this period. Also in this period, the “health imperative”—as it is now called—was idealized^20^, based on the idea of improving individual health, taking this as a goal in and of itself, reducing health to the absence of disease, while placing responsibility with individuals for managing their health problems.

However, a greater emphasis on social determinants of health goes beyond the increase in scientific publications and research groups. Health observatories and the Sustainable Development Goals, according to which various countries are committed to achieving goals to improve their populations’ life conditions and health profiles, are highly relevant in developing policies for a fairer world.

While there is no doubt that health is historically and socially determined, at the same time, this knowledge is insufficient to underpin studies and influence policies, public health management or health care, what is the role of studies on social determinants of health, today? Do studies that simply replicate these associations without proposing actions or policies to reduce injustice add to the current knowledge on the topic? Particularly in Brazil—which since the 2014 presidential election has experienced a crisis that resulted in the 2016 institutional coup, whose negative consequences are expressed in several health indicators in the short, medium and long term^21,22^—how could these studies contribute to addressing and reversing this picture?

The answer to this question involves the knowledge that health is, rather than the absence of disease, the ability to live a significant life, despite any limitations^4^, and that health is produced collectively. This perspective could be employed in each study to propose effective public policies to improve the population’s health^22^. We contend that reiterating the evidence for social inequalities in health without formulating clear strategies to address them operates as a social buffer, and eventually naturalizes injustice^23^. Uncritical repetition of a social fact leads to its naturalization; that is, to the understanding that unfair and avoidable social inequalities in health are intrinsic to societies.

In other words, the theoretical frameworks of studies in the area must recognize that social determinants of health operate via both contemporary social configurations and historical processes; and, consequently, they should make explicit recommendations to reduce inequalities. This proposition, however, is not recent and is visible in the clashes between a social determinants of health approach and a focus on the social determination of the health-disease-care process. The former allows for the investigation of variables without articulating social and historical processes underpinning them, whereas the second understands the problem as a historical process and calls into question the causes of the “causes of causes”—the modes of social organization and their consequences for people’s health^23^. Studies must answer research questions that are based on the reality in which a population lives. They must also consider the historical processes that give rise to such a reality.

Not considering social and historical processes is a theoretical failure that leads to a methodological flaw: neglecting a deep analysis when interpreting associations and models that include indicators of social determinants of health. In an epidemiologic study, a variable is only a descriptor of a condition or a situation. By not appropriately locating this condition or situation, researchers may end up taking study results at face value. The view that variables are objective measures is nothing more than a methodological sleight of hand, which does not justify, and should not lead to reification, of the context under study^24^.

An editorial recently published in *The Lancet Public Health* reminds us that poverty and other forms of oppression, as well as their consequences for the health-disease-care process, are political choices^25^. Scientific investigation guided by a focus on the determination of health rather than the determinants of health will influence actions that may foster and promote social justice and, consequently, improve the populations’ health.
