# Supplementary material for: Whither social determinants of health?
Source: Rev Saude Publica. 2020 Jan 23;54:15. doi: 10.11606/s1518-8787.2020054001618 (PMC6986865; doi:10.11606/s1518-8787.2020054001618)
Supplement: Translation

# Translation: Para onde seguir com a pesquisa em determinantes sociais da saúde?

**Authors:** Fúlvio Borges Nedel, João Luiz Bastos

**Affiliations:** I Brasil Universidade Federal de Santa Catarina. Departamento de Saúde Pública. Florianópolis, SC, Brasil

**Keywords:** Determinantes Sociais da Saúde, Saúde Pública, Disparidades nos Níveis de Saúde, Iniquidade Social, Pesquisa

## Abstract

Este comentário crítico retoma o debate sobre a determinação social da saúde e da doença. Seu principal argumento é o de que, se por um lado, são desnecessárias novas pesquisas que procurem conferir maior consistência aos resultados que fundamentam essa interpretação, por outro, é escassa, nesses estudos, uma análise pautada pela noção de que a pobreza e outras formas de opressão são escolhas políticas da sociedade, que devem ser situadas historicamente. Essa noção deve orientar a pesquisa e o debate na área, sob pena de que as associações encontradas supostamente para denunciar a injustiça terminem por naturalizá-la. A pesquisa pautada por esses princípios pode superar o alcance limitado de abordagens multicausais do tipo caixa-preta, que não analisam as inter-relações entre os determinantes e pouco contribuem para a construção de sociedades saudáveis.

## Para onde seguir com a pesquisa em determinantes sociais da saúde?

A noção de que a saúde é determinada socialmente, isto é, que a saúde do indivíduo depende da sociedade em que ele vive, não é nova e já se pode perceber em textos hipocráticos, como *Ares, águas e lugares*. Tal ideia volta a operar no mundo ocidental a partir do trabalho de Ramazzini sobre *As doenças dos trabalhadores*, ao final do século XVII. Na primeira metade do século XIX, com os trabalhos de Alexandre-Louis, Villermé, Engels e sobretudo com o movimento da medicina social de Virchow e outros, vai-se estruturando o conhecimento em torno da ideia de que as condições de saúde, adoecimento e morte das pessoas (e consequentemente das populações) dependem das condições de vida, que dependem, por sua vez, das condições sociais de reprodução da vida^1^.

Deve-se notar que tal conhecimento (e poucas associações serão tão evidentes quanto as relatadas por Villermé de que metade dos filhos dos patrões chegavam aos 21 anos, enquanto metade dos filhos dos operários não alcançavam os dois anos de idade) não pressupõe o estabelecimento de um agente etiológico da doença. Entretanto, convive com dois problemas intimamente relacionados: (1) o desejo de encontrar explicações simples para algumas questões, como que cada doença tenha uma única causa, mais ou menos específica – explicação que ainda se busca, agora trasladada à epidemiologia molecular e genética^2^; e (2) está no cerne de uma disputa ideológica fundamental que nos remonta, pelo menos, a Chadwick^3^ e se traduz pela indagação “deve-se mudar a organização social para promover saúde?”

Assim, em meio ao capitalismo crescente, o advento da bacteriologia ao final do século XIX serviu para eludir as propostas de mudanças nas cidades e na sociedade apresentadas pelos então defensores das teorias miasmáticas de etiologia das doenças^3^. Pode-se dizer que, quando nos limpamos das teorias miasmáticas, junto à água desse banho o capitalismo daquele momento aproveitou para jogar fora a criança da determinação social da saúde. Porque o entendimento de que a saúde é socialmente determinada traz o imperativo da mudança social para a melhoria das condições de saúde da população. Se desejamos uma sociedade com menos desigualdades injustificáveis, precisamos construir uma sociedade mais justa, com menos injustiças sociais. Para isso, o avanço no conhecimento biomédico é necessário, mas insuficiente^4^. Temos então, além do mais, uma questão ética que é igualmente central. O problema não é simplesmente que haja diferenças em saúde. Elas existirão, posto que as pessoas e as sociedades são diferentes, e não devem ser a epidemiologia ou a saúde pública instrumentos de homogeneização cultural; o nosso foco é nas diferenças de saúde injustas^5^.

Nesse sentido, é notável a definição de saúde pública de Winslow, no início do século XX^6^, em plena vigência da explicação unicausal da doença, ainda que mais ou menos suavizada na concepção de “tríade ecológica”^7^. Bacteriologista de formação e fundador do Departamento de Saúde Pública da Escola de Medicina da Universidade de Yale, nos Estados Unidos da América, Winslow foi o propositor de uma das definições de saúde pública mais citadas até hoje. Numa publicação em que o autor discute o caráter multidisciplinar da saúde pública, além das amplas atribuições dos seus profissionais, Winslow arrisca a seguinte definição para a área: “A saúde pública é a ciência e a arte de prevenir doenças, prolongar a vida e promover saúde física e eficiência, através de esforços comunitários organizados para o saneamento do meio ambiente, controle de infecções comunitárias, educação do indivíduo em princípios de higiene pessoal, organização do serviço médico e de enfermagem para o diagnóstico precoce e tratamento preventivo da doença e desenvolvimento da maquinaria social que garantirá a todos os indivíduos da comunidade um padrão de vida adequado para a manutenção da saúde” ^6^.

Essa definição não só delimita a saúde pública a partir da saúde – e não da doença –, como recupera a noção e inclusive a retórica presente no *Manifesto da Medicina Social* de Virchow de que a sociedade pode se organizar de modo a garantir um padrão de vida adequado à manutenção da saúde de seus indivíduos^8^. Apresenta, além disso, uma aguda distinção entre conceitos que foram, somente em décadas subsequentes, amplamente debatidos e refinados na área, tais como os de prevenção de doenças e de promoção da saúde^9^. Winslow estabelece estreita vinculação das condições populacionais de saúde, doença e bem-estar com aspectos de ordem social, econômica e política: os serviços e a maquinaria social a que o autor se refere. Arriscamos argumentar, entretanto, que essa definição ganhou popularidade mais em função de sua dimensão bacteriológica – que permitiu ao *establishment* a ênfase no ataque à doença dos indivíduos e o entendimento de que a promoção da saúde é apenas parte da prevenção de doenças – do que por sua tentativa de retomar a noção de determinação social da saúde.

Assim, apesar de definições como a de Winslow, a retomada de perspectivas que privilegiam uma relação de dependência entre as formas de organização social e a saúde dos indivíduos (em seu conjunto) coexistiu com uma reafirmação do pensamento biomédico sobre os problemas de saúde. A partir da segunda metade do século XX, em meio à transição epidemiológica e após a experiência de duas guerras mundiais, do nazismo e do fascismo, do Plano Marshall e do macarthismo^10^, torna-se hegemônica a concepção causal da caixa-preta^11^. Essa proposta pretende-se multicausal, mas logo se revela unicausal ao explicar a função de cada fator como se atuasse isoladamente, numa análise simplista que não se preocupa com as relações entre os fatores e, menos ainda, com suas causas (as “causas das causas”^12^)^13^. Com efeito, multicausalidades descontextualizadas (ou “teias sem aranha” – isto é, redes causais sem causadores, nas palavras de Nancy Krieger^13^) que haviam sido desenvolvidas décadas antes ganharam forma e inúmeros adeptos, sendo por vezes as abordagens hegemônicas para a explicação da origem de doenças e a proposição de estratégias para seu enfrentamento. Nessas vertentes biomédicas, predominou a ênfase em características clínicas (colesterol elevado, hipertensão arterial, sedentarismo etc.) como elementos produtores de morbimortalidade, todos flutuando num verdadeiro vácuo social e pouco situados de um ponto de vista histórico. Em análises com tal base teórica, os determinantes sociais da saúde, mesmo quando incluídos, têm pouco papel a cumprir, por maior que seja a significância estatística que alcancem em modelos multinível ou de equações estruturais.

São dignas de nota as contribuições teóricas e empíricas que demonstraram a insuficiência da caixa-preta^14-16^. Insuficiência dada, não é demais ressaltar, não apenas por uma abordagem biomédica estrita, mas pela indisposição a uma análise mais complexa, que inclua a relação entre os fatores analisados e suas determinações, pelo menos, no modelo teórico do estudo. Isso significa que a abordagem comportamentalista e outras que eventualmente incluam variáveis indicadoras da condição social ou “de contexto” tampouco resolvem o problema, se não derem conta dessa limitação de base.

As crises associadas aos embates entre esses distintos estilos de pensamento em saúde pública^17^, mas especialmente às contradições reveladas pela distância entre as abordagens ainda mais positivistas e a definição da saúde pública de Winslow^6^, geraram consternação em alguns profissionais da área. Fazendo recurso a um tribunal de justiça imaginário em um artigo de 1997, Shy^18^ serve de “testemunha de acusação” de que a epidemiologia não atende aos interesses últimos da saúde pública. Em meio a esse cenário, criticou a epidemiologia biomédica e hegemônica, argumentando contrariamente à insistência em fatores de ordem “micro” e majoritariamente clínicos, que pouco contribuem para o desenho de intervenções efetivas para a melhora da saúde das populações. Tal insistência, aliada a uma descontextualização social e histórica, teria limitado o estudo das forças políticas, econômicas e culturais enquanto determinantes de padrões populacionais de saúde e doença^4,18^.

Há que se observar, entretanto, que isso acontece *pari passu* ao aumento de publicações^19^ e grupos de pesquisa sobre desigualdades e determinantes sociais em saúde, especialmente nas duas últimas décadas. Foi nesse período que se observou um aumento expressivo na quantidade de publicações^19^ que se preocupam em dar maior consistência a um conhecimento que, conforme apontamos acima, já está bem estabelecido. É também nesse período que se construiu o que agora tem sido denominado “imperativo da saúde”^20^, que, sob o pretexto de melhorar a saúde dos indivíduos, a coloca como o objetivo último a ser atingido, a reduz novamente à ausência de doenças e aprofunda a culpabilização do indivíduo e sua responsabilidade na gestão de seus problemas de saúde.

No entanto, o maior reconhecimento dos determinantes sociais da saúde vai além do aumento dessas publicações científicas e dos grupos de pesquisa. Os observatórios em saúde e os Objetivos de Desenvolvimento Sustentável, em que os Estados de diferentes países se comprometem a alcançar metas indicadoras de melhoras na situação de vida e saúde de suas populações, são de grande relevância prática para o desenvolvimento de políticas para um mundo mais justo.

Assim, em tal situação em que não há mais dúvida de que a saúde é determinada histórica e socialmente, mas, ao mesmo tempo, de que isso não é suficiente para que essa relação se estabeleça como base teórica dos estudos e orientadora das políticas, gestão ou assistência em saúde, qual o papel dos estudos sobre determinantes sociais em saúde? Contribuem para o conhecimento os que replicam essas associações sem propor ações ou políticas para a redução da injustiça? Particularmente no caso do Brasil – que desde a eleição presidencial de 2014 vive uma crise que só se aprofundou com o golpe institucional de 2016 e cujas graves consequências se expressam na saúde com a piora de vários indicadores em curto, médio e longo prazo^21,22^ –, como esses estudos poderiam contribuir para o enfrentamento e a reversão desse quadro?

A resposta a essa indagação passa por aplicar, em cada estudo, o conhecimento acumulado (i.e., de que a saúde é a capacidade de viver uma vida significativa, apesar de suas limitações^4^, não a ausência de doença, e de que sua produção é coletiva) para a proposição de políticas públicas concretas de melhoria da saúde da população analisada^22^. De nossa parte, entendemos que a reiterada denúncia das desigualdades sociais em saúde desacompanhada de proposições para seu enfrentamento exerce uma função de tampão social e naturalização da injustiça^23^, pois a repetição acrítica de um fato social leva à sua naturalização, ou seja, ao entendimento de que aquele fato é inerente às sociedades.

Em outras palavras, o referencial teórico dos estudos na área deve necessariamente reconhecer que os determinantes sociais da saúde operam em processos históricos de distintas durações e, consequentemente, fazer recomendações concretas para a redução das desigualdades. Tal proposição não é recente, entretanto, e pode ser identificada nos embates entre a abordagem sobre determinantes sociais da saúde e determinação social do processo saúde-doença-atenção. Enquanto a primeira permite a investigação de variáveis sem que processos sociais e históricos sejam com elas articulados, a segunda entende o problema como um processo histórico e coloca em questão as causas das “causas das causas” – os modos de organização social e suas consequências sobre a saúde das pessoas^23^. Os estudos devem responder a perguntas de pesquisa que se ancorem na realidade em que vive a população estudada e percebam o processo histórico que o constrói.

Não perceber o processo histórico é uma falha teórica que desemboca numa falha metodológica: a falta de retorno à categoria de análise ao interpretar o resultado das associações e modelos com variáveis descritoras dos determinantes sociais. Num estudo epidemiológico, uma variável é apenas um descritor de uma situação. Não retomar a referida situação implica interpretar os resultados do estudo pelo seu valor de face. O fato de as variáveis serem medidas objetivas (positivas, positivistas) não é mais que um artifício metodológico, que não justifica nem deve conduzir à reificação da situação estudada^24^.

Um editorial recentemente veiculado no periódico *The Lancet Public Health* lembra que a pobreza e outras formas de opressão, bem como suas consequências para o processo saúde-doença-atenção, nada mais são do que escolhas políticas^25^. A pesquisa que se paute por esses princípios – o estudo da determinação e não dos determinantes – terá um maior potencial de influenciar ações com impacto positivo sobre a democracia e a justiça social e, consequentemente, produzir repercussões positivas para a saúde das populações.
